# Specificities and Efficiencies of Primers Targeting *Candidatus* Phylum Saccharibacteria in Activated Sludge

**DOI:** 10.3390/ma11071129

**Published:** 2018-07-03

**Authors:** Ryota Takenaka, Yoshiteru Aoi, Noriatsu Ozaki, Akiyoshi Ohashi, Tomonori Kindaichi

**Affiliations:** 1 Department of Civil and Environmental Engineering, Graduate School of Engineering, Hiroshima University, 1-4-1 Kagamiyama, Higashihiroshima 739-8527, Japan; m171656@hiroshima-u.ac.jp (R.T.); ojaki@hiroshima-u.ac.jp (N.O.); ecoakiyo@hiroshima-u.ac.jp (A.O.); 2 Department of Molecular Biotechnology, Graduate School of Advanced Sciences of Matter, Hiroshima University, 1-3-1 Kagamiyama, Higashihiroshima 739-8530, Japan; yoshiteruaoi@hiroshima-u.ac.jp

**Keywords:** *Candidatus* Saccharibacteria, primer evaluation, primer specificity, quantitative PCR, probe

## Abstract

*Candidatus* Saccharibacteria is a well-described candidate phylum that has not been successfully isolated. Nevertheless, its presence was suggested by 16S rRNA gene sequencing data, and it is frequently detected in natural environments and activated sludge. Because pure culture representatives of *Candidatus* Saccharibacteria are lacking, the specificity of primers for the determination of their abundance and diversity should be carefully evaluated. In this study, eight *Candidatus* Saccharibacteria-specific primers were selected from previous studies and evaluated for their coverage against a public database, annealing temperature of the combined primer sets, as well as their utilization to determine the detection frequencies and phylogenetic diversity by cloning analysis, and in quantification by quantitative polymerase chain reaction (PCR). Among the eight primers, four primers (TM7314F, TM7580F, TM7-910R, and TM7-1177R) showed high coverage. Cloning analysis showed that four primer sets (TM7314F and TM7-910R, TM7314F and TM7-1177R, TM7580F and TM7-910R, and TM7580F and TM7-1177R) yielded high detection frequencies for *Candidatus* Saccharibacteria in activated sludge from a wastewater treatment plant in Higashihiroshima City, Japan. Quantitative PCR results indicated that the primer set containing TM7314F and TM7-910R was superior for the specific detection of *Candidatus* Saccharibacteria in activated sludge.

## 1. Introduction

The specific detection of microorganisms of interest from complex multispecies microbial communities in the natural environment and engineering systems such as activated sludge is crucial to understanding the presence and activity of key microorganisms. However, traditional cultivation and microscopic techniques are not adequate because most microorganisms present in the natural environment and engineering systems cannot be cultivated. In activated sludge, only a small fraction (1% to 10%) of total microorganisms have been isolated and characterized to date [1]. Therefore, culture-independent molecular biology techniques have been widely applied for the specific detection of key microorganisms and the microbial community structures. Among the specific detection techniques, fluorescence in situ hybridization (FISH) and quantitative PCR (qPCR) are essential to detect specific microorganisms in complex microbial communities, although high throughput sequencing techniques are rapidly being developed. In FISH and qPCR techniques, the specificity of probe (for FISH) and primers (for qPCR) is the critical parameter for detecting specific microorganisms, including candidate division microorganisms, which have not been successfully isolated but their presence was suggested using 16S rRNA sequencing [2,3,4]. Thus, the specificity of probes and primers for the specific detection of candidate division microorganisms should be carefully evaluated because of the lack of pure culture representatives. Candidate division microorganisms have recently been classified into the candidate phyla radiation (CPR) [5], an expansion of the tree of life. The CPR comprises >15% of all bacterial diversity and potentially contains >70 phyla [6].

*Candidatus* Saccharibacteria (formerly known as candidate division TM7, hereafter referred to as Saccharibacteria) is a well-described candidate phylum and has been frequently detected in natural environments [7], the human microbiome [8,9], and activated sludge [10,11,12]. The phylum was recently assigned the name Saccharibacteria, owing to their sugar metabolisms [11]. Within the phylum, three subdivisions have been proposed according to the 16S rRNA gene sequences [10]. Members of subdivision one show filamentous morphotypes, whereas members of subdivisions two and three show non-filamentous morphotypes (i.e., rods or cocci). Filamentous bacteria are the main contributors to the bulking issues in wastewater treatment as their excessive growth causes poor settling of biomass in clarifiers. Although Saccharibacteria, including those with filamentous morphotype, have been frequently detected in activated sludge by FISH analysis, the relationship between their abundance and bulking issues is unclear [12]. Additionally, Saccharibacteria have been implicated in human mucosal diseases based on their high abundance in certain oral bacterial communities [13]. Nevertheless, despite their importance, little is known about their phylogeny and physiology, especially about those related to human diseases.

To date, several Saccharibacteria-specific primers and probes based on 16S rRNA gene sequences have been reported; however, some showed low specificities and produced false-positive signals [14,15]. These observations suggested an overestimation of Saccharibacteria abundance in environmental and human samples. Therefore, evaluation of primer and probe specificity is critical for the accurate detection, as well as evaluation, of Saccharibacteria isolation (i.e., pure culture). Indeed, Soro et al. were the first to report a pure culture of a Saccharibacteria species, and Saccharibacteria-specific primers and probes were used to confirm the quality of the isolation [8].

In this study, we evaluated the specificity of previously reported Saccharibacteria-specific primers. Eight specific primers were chosen from the literature, and the coverage of each primer was reevaluated using a 16S rRNA database. The annealing temperature of four primer sets that displayed high primer coverage was subsequently determined. Their specificity for Saccharibacteria sequences was evaluated by cloning and sequence analysis of 16S rRNA genes from activated sludge. The four primer sets were also used to evaluate Saccharibacteria 16S rRNA gene copies in an activated sludge sample by qPCR. It should be noted that we did not include the primer set reported by Ferrari et al. [7] because they have already shown the high coverage and specificity for Saccharibacteria.

## 2. Results and Discussion

### 2.1. Assessment of Selected Saccharibacteria-Specific Primers

Eight Saccharibacteria-specific primers were selected from previous studies [10,13,16,17] and evaluated in this study (Table 1). The specificity of the primers was evaluated in silico in terms of sequence coverage by aligning them with Saccharibacteria sequences available in the SILVA ribosomal RNA gene database [18] and counting the mismatches; primers with >95% average coverage were selected for further analyses. Among the eight primers, TM7314F, TM7580F, TM7-910R, and TM7-1177R showed the highest coverages in all sequence positions (Figure S1). Although TM7580F is degenerated (Y) at the second position (Figure S1B), most of Saccharibacteria sequences from the database had C (1286) as compared to T (16) in the position. Therefore, a modified primer for TM7580F, 5′-ACTGGGCGTAAAGAGTTGC-3′, may also be used for the specific detection of Saccharibacteria. In this study, these four primers were further evaluated.

### 2.2. Determination of Optimal Annealing Temperatures

**Figure 1:**
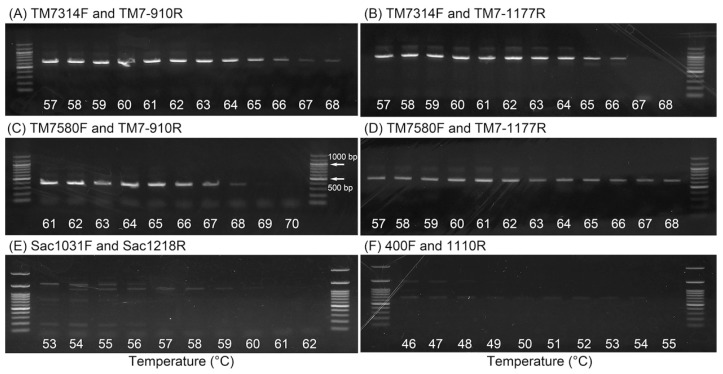
Agarose gel electrophoresis pattern of the PCR products using the following primer sets: TM7314F and TM7-910R (**A**), TM7314F and TM7-1177R (**B**), TM7580F and TM7-910R (**C**), TM7580F and TM7-1177R (**D**), Sac1031F and Sac1218R (**E**), and 400F and 1110R (**F**).

Because the optimal annealing temperature is dependent on the primers and DNA samples, the annealing temperature for four Saccharibacteria-specific primer sets was determined using DNA extracted from activated sludge and temperature-gradient PCR from 57 to 70 °C. No DNA bands were observed in gel electrophoresis from PCR amplification with the other two primer sets (Sac1031F and Sac1218R; 400F and 1110R) at the proposed annealing temperature (60 °C for both primer sets; Figure 1E,F and Figure S2). In this study, the optimal annealing temperature was determined based on the decreasing amplicon band intensity on agarose gel (Figure 1), and decreasing concentration of PCR products with increasing annealing temperature (Figure S2). The optimal annealing temperatures of the four primer sets are listed in Table 2. These annealing temperatures were higher than the original primer melting temperatures (T_m_) [10,16].

### 2.3. Evaluation of Primer Specificity by Cloning Analysis

**Figure 2:**
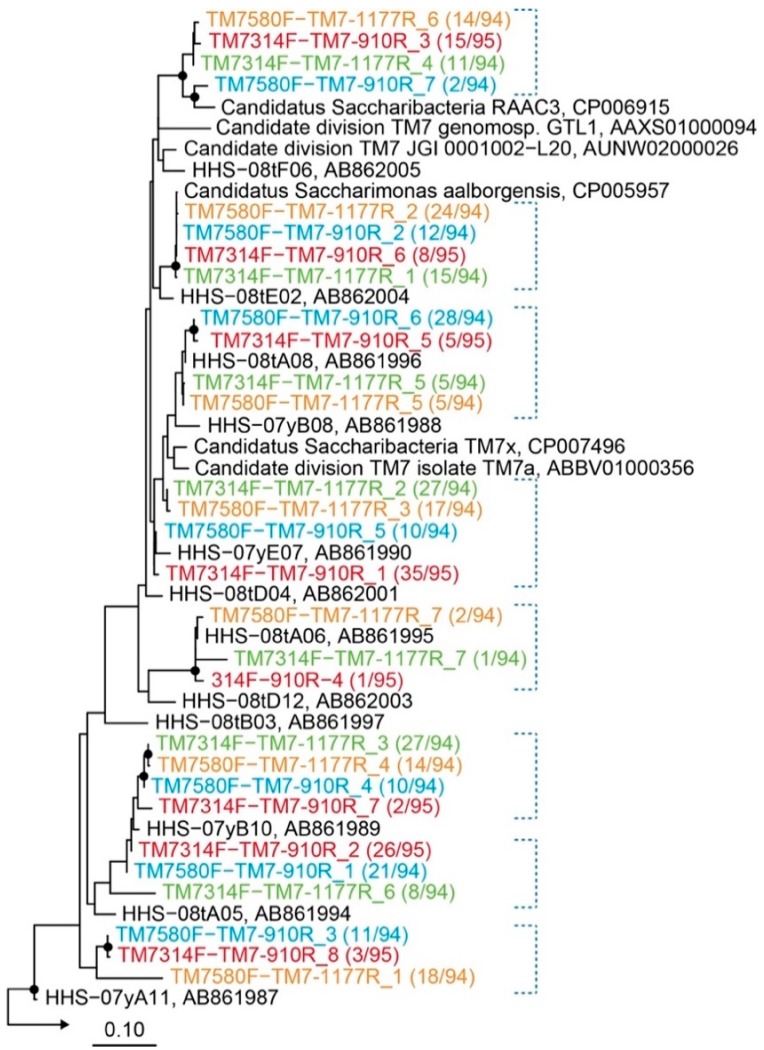
Maximum-likelihood phylogenetic tree of Saccharibacteria-related operational taxonomic units (OTUs) obtained from activated sludge. Colored OTUs represent OTUs obtained from different primer sets. Numbers in parentheses indicate the frequency of identical clones analyzed from each library. The scale bar represents the number of nucleotide changes on each position. Filled circles at the nodes represent bootstrap support of >90% obtained from 1000 resamplings. Blue dotted lines indicate identical clones detected with different primer sets. Five *Thermotoga* sequences were used as an outgroup. Black sequences labelled as HHS indicate OTUs obtained from a previous study [12]; other black sequences are reference sequences from the database.

To investigate the specificity and phylogenetic diversity of Saccharibacteria in activated sludge, PCR, cloning, and sequence analyses were conducted using activated sludge samples. Four clone libraries were constructed from 16S rRNA genes amplified using the four primer combinations, and 94 to 96 clones were randomly chosen from each library and sequenced. No chimeric sequences were observed and the specificity for Saccharibacteria was 100% for the four clone libraries. Microorganisms affiliated with other phyla were not detected by a BLAST search [19]. These results clearly demonstrated that the four primer sets were highly specific for Saccharibacteria. The clones were subsequently grouped into operational taxonomic units (OTUs) based on >97% sequence identity. Seven to eight OTUs were obtained from each clone library (29 OTUs in total), and a phylogenetic tree was constructed to include Saccharibacteria-related OTUs (Figure 2). The detailed phylogenetic tree and relatives are shown in Figure S3 and Table S1. The OTUs obtained formed eight clusters (blue dotted lines in Figure 2). The four primer sets detected nearly identical clones. Each of the eight clusters contained at least three OTUs (most had four OTUs) obtained from different primer sets, indicating that a similar phylogenetic diversity of OTUs was detected using the four primer sets. Most of the clusters were closely related to OTUs obtained from activated sludge samples from the same facility analyzed in a previous study (reference sequences [black] labelled as HHS-clones in Figure 2) [12]. This observation suggested that the dominant members of Saccharibacteria in activated sludge from the facility did not change over a long period of time. Other Saccharibacteria reference sequences were also included in the tree; these Saccharibacteria were found in activated sludge as well as in the oral cavity, both being rich in organic matters and having fluctuating anaerobic and aerobic environments.

### 2.4. Evaluation of Primer Specificity by qPCR

Due to the high specificity for Saccharibacteria in activated sludge, the four primer sets were used to evaluate the quantity of Saccharibacteria 16S rRNA copies and amplification efficiencies by qPCR using three types of template DNA: plasmid DNA (with known copy number of Saccharibacteria 16S rRNA gene), DNA extracted from activated sludge (unknown copy number of Saccharibacteria 16S rRNA gene), and control DNA (contained the 16S rRNA gene of *Hydrogenophaga* sp.).

**Figure 3:**
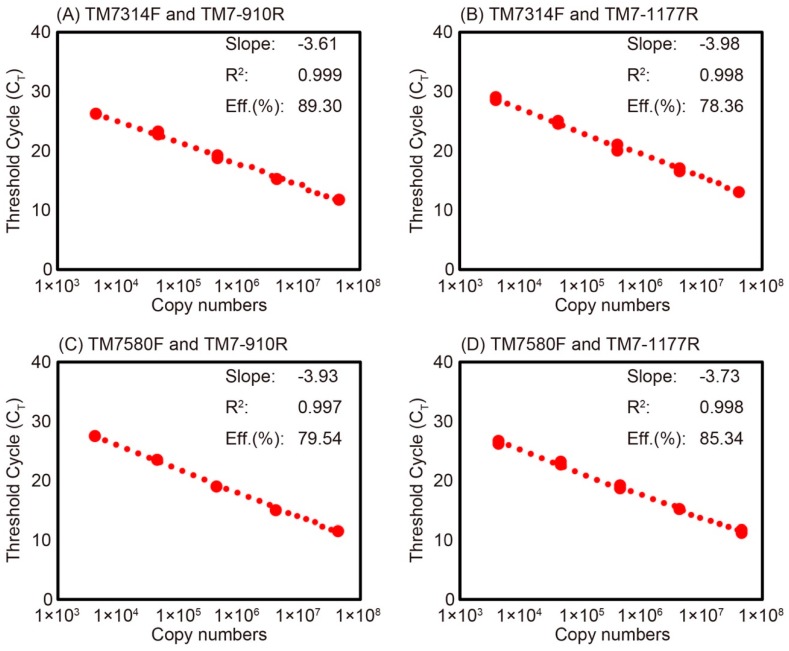
Standard curves of Saccharibacteria qPCR for the measurement of activated sludge samples using 10-fold serial dilutions of plasmid DNA carrying Saccharibacteria 16S rRNA genes and the four primer sets: TM7314F and TM7-910R (**A**); TM7314F and TM7-1177R (**B**); TM7580F and TM7-910R (**C**); and TM7580F and TM7-1177R (**D**). The slope, coefficient of determination (R^2^), and amplification efficiency are also shown in the figures.

**Figure 4:**
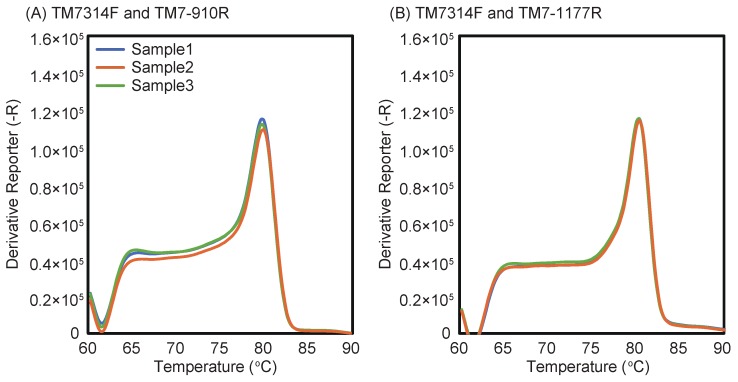

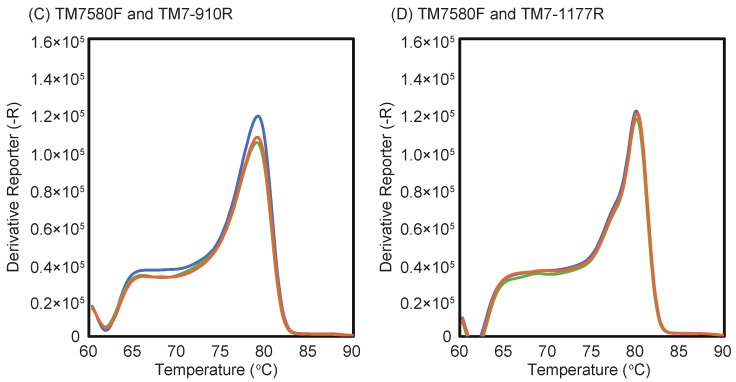
Melting curves of qPCR reactions using activated sludge samples and the four primer sets: TM7314F and TM7-910R (**A**); TM7314F and TM7-1177R (**B**); TM7580F and TM7-910R (**C**); and TM7580F and TM7-1177R (**D**).

Standard curves for the four primer sets were constructed from a series of 10-fold dilutions of plasmid DNA carrying partial 16S rRNA gene of Saccharibacteria, ranging from 1.17 × 10^3^ to 6.15 × 10^7^ copy numbers (Figure 3). The standard curves of primer sets with TM7-910R showed higher amplification efficiencies (Figure 3A,C), whereas those with TM7-1177R showed <90% amplification efficiencies (Figure 3B,D). The melting curves using DNA extracted from activated sludge for the four primer sets indicated the presence of specific amplification products at around 80 °C (Figure 4). The standard curves and melting curves for the four primer sets using plasmid DNA carrying Saccharibacteria 16S rRNA genes are shown in Figures S4 and S5.

The accuracy of the copy number quantification of Saccharibacteria 16S rRNA gene was confirmed by qPCR. First, a dominant clone recovered by all four clone libraries was selected, and plasmid DNA was extracted. These four plasmids were carrying inserts of different lengths owing to the different primer sets used. The plasmid copy numbers were calculated from the concentration of extracted plasmids (‘Copy number of plasmid’ in Table 3), based on a method described previously [21] and the following equation:

(1) $$\;Copy\;number=\frac{A}{\left(B+3931\right)/660}\times 6.02\times 10^{14}$$

where *Copy number* is in copies μL^–1^, *A* denotes plasmid concertation (ng μL^–1^), *B* denotes the length of the PCR product (bp), 3931 denotes the length of vector (pCR2.1-TOPO), 660 denotes the average molecular weight of one base pair (g mol^–1^ bp^–1^), and 6.02 × 10^14^ denotes the Avogadro number (mol^–1^) with a unit conversion (gram to nanogram, i.e., ×10^–9^).

The calculated copy numbers were verified by qPCR. The measured values were 1.3- to 2.1-fold higher than the calculated values. In addition, a plasmid carrying the 16S rRNA gene of *Hydrogenophaga* sp., which has two to four sequence mismatches versus the primers (Table 4), was also quantified with the four Saccharibacteria primer sets as the control. The quantified copy numbers were below detection limits (1.2 × 10^3^ to 6.2 × 10^3^ copies). Therefore, primer pairs can be considered specific to the Saccharibacteria and suitable for qPCR when the threshold is set to the detection limits mentioned above.

Next, the copy number of Saccharibacteria in an activated sludge sample was quantified using the four primer sets (Table 3). The measured copy numbers varied from 6.8 × 10^6^ to 6.7 × 10^7^ copies per gram of mixed liquor suspended solids (MLSS). The use of TM7580F resulted in one order of magnitude higher values, indicating overestimation. These results suggested that the TM7314F and TM7-910R, and TM7314F and TM7-1177R primer sets are superior for the specific detection of Saccharibacteria in activated sludge. These two primer sets may also be used for tRFLP analysis [22] and 16S rRNA gene amplicon analysis with long-read high throughput sequencing (e.g., Pacific Biosciences PacBio, Oxford Nanopore). All eight primers listed in Table 1 may also be used as FISH probes, because they have high specificity for Saccharibacteria. Indeed, the TM7305 probe and TM7905 probe [10] were widely used in FISH analysis. Since shorter PCR products are preferred for accurate quantification by qPCR, the use of TM7314F and TM7-910R is more suitable for the specific detection of Saccharibacteria with qPCR.

## 3. Materials and Methods

### 3.1. Primer Selection and Evaluation

Saccharibacteria-specific primers were selected from the literature [10,16] to assess their specificity (Table 2). The specificity was evaluated by counting the primer/target mismatches using the Saccharibacteria sequences in the SILVA small subunit (SSU) Reference non-redundant (NR) release 132 database [18] and the ARB software (version 6.0.4) [20], and displayed as coverage. Three categories were set: >90%, >75%, and >50%.

### 3.2. Activated Sludge Samples

Activated sludge was collected four times between April 2016 and March 2018 from the aeration tank of a municipal wastewater treatment plant in Higashihiroshima City, Japan [23]. The plant has been in stable operation for several years and a relatively high abundance of Saccharibacteria [12]. The sludge samples were stored at −20 °C before being analyzed in the experiments.

### 3.3. DNA Extraction and PCR Amplification

DNA was extracted from the activated sludge samples using a FastDNA spin kit (MP Biomedicals, Irvine, CA, USA). The extracted DNA was used for PCR amplification using Emerald Amp PCR Master Mix (Takara Bio Inc., Kusatsu, Japan) with six combinations of forward and reverse primers listed in Table 2. The PCR mixture (25 μL) consisted of 12.5 μL Emerald Amp PCR Master Mix (Takara Bio Inc.), 0.5 μL each of the forward and reverse primers (25 μM), 0.5 μL template DNA (5–10 ng), and 11 μL molecular biology grade water (Roche Applied Science, Mannheim, Germany). The PCR conditions were as follows: 3 min of initial denaturation at 95 °C, followed by 30 cycles of 30 s at 95 °C, 30 s at annealing temperature, and 30 s at 72 °C. The final extension was performed for 5 min at 72 °C. The optimal annealing temperature of each primer set was evaluated by temperature-gradient PCR, and the PCR products were subjected to 1% agarose gel electrophoresis and/or purification using Agencourt AMPure XP (Beckman Coulter, Brea, CA, USA) followed by the determination of DNA concentrations using a Qubit dsDNA HS Assay Kit (Thermo Fisher Scientific, Waltham, MA, USA), according to the manufacturer’s protocols.

### 3.4. Cloning, Sequencing, and Phylogenetic Analysis

Purified PCR products were ligated into the TOPO vector. Clone libraries were constructed by transforming One Shot *E. coli* cells with the ligation product according to the manufacturer’s instructions (TOPO pCR2.1 Cloning Kit; Life Technologies, Carlsbad, CA, USA). A total of 96 clones per primer pair were picked and the plasmid-carried partial 16S rRNA genes were amplified by colony PCR with M13F and M13R primers. Plasmid purification was carried out using a FastGene Plasmid Mini Kit (Nippon Genetics Co., Ltd., Tokyo, Japan). The partial 16S rRNA genes obtained were sequenced by Takara Bio Inc). All sequences were checked for chimeric artifacts using the ChimeraSlayer program [24]. The sequences were aligned with the Integrated Aligners program in the ARB software [20]. Sequences with 97% or higher similarity were grouped into OTUs using the distance matrix methods with similarity correction in the ARB software [20]. A phylogenetic tree was constructed using the maximum likelihood (RAxML) methods using default settings in the ARB software with the SILVA SSU Ref NR release 132 database [18]. A bootstrap resampling analysis for 1000 replicates was conducted using the ARB software to estimate the confidence of the tree topology. The 16S rRNA gene sequence data of OTUs obtained in this study were deposited in the GenBank/EMBL/DDBJ databases under accession numbers LC380997–LC381025 and LC388572. The specificity for Saccharibacteria-related clones was evaluated for each primer set.

### 3.5. Real-Time Quantitative PCR

To evaluate the suitability of the four primer sets for the quantification of Saccharibacteria, the copy number of Saccharibacteria 16S rRNA genes was determined by qPCR. Plasmid DNA (with known copy number of Saccharibacteria 16S rRNA gene), DNA extracted from activated sludge (unknown copy number of Saccharibacteria 16S rRNA gene), and control DNA (containing the 16S rRNA gene of *Hydrogenophaga* sp.) were used for the evaluation. The qPCR was performed using the StepOne Real Time PCR system (Thermo Fisher Scientific) according to the manufacturer’s instructions. The qPCR mixture (20 μL) consisted of 10 μL Fast SYBR Green Master Mix (Thermo Fisher Scientific), 1 μL each of the forward and reverse primers (10 μM), 2 μL template DNA (5–10 ng), and 6 μL PCR grade water (Roche Applied Science). The qPCR conditions were as follows: initial activation at 95 °C for 20 s, followed by 40 cycles at 95 °C for 3 s, and annealing for 30 s. The annealing temperature of each primer set is listed in Table 2. All qPCR runs were performed alongside non-template controls in triplicate. Standard curves were constructed from 10-fold dilutions of plasmid DNA (pCR2.1-TOPO, Thermo Fisher Scientific) carrying partial Saccharibacteria 16S rRNA genes retrieved from a clone library described in section 3.4 with a known copy number. The plasmid concentrations were measured using a Qubit dsDNA HS Assay Kit (Thermo Fisher Scientific). Plasmid DNA of a known copy number carrying the 16S rRNA gene of *Hydrogenophaga* sp., which has two to four sequence mismatches versus the Saccharibacteria primers, was used as the negative control (Table 4).

## 4. Conclusions

In this study, four primer sets specific to Saccharibacteria were evaluated for five aspects: (1) the primer coverage, (2) re-measurement of annealing temperature for the primer sets, (3) specificity by cloning analysis, (4) evaluation of standard and melting curves of the primer sets, and (5) quantification of Saccharibacteria in an activated sludge sample. The evaluation of primer coverage and specificity by cloning showed that the four primer sets had high specificities. However, TM7580F showed a potential for overestimation by standard curve and melting curve analyses, as well as Saccharibacteria quantification in activated sludge. From the qPCR results, we concluded that the use of the TM7314F and TM7-910R primer set at an annealing temperature of 64 °C was optimal for the specific detection of Saccharibacteria in activated sludge. The efficiency of the TM7314F and TM7-910R primer set to detect Saccharibacteria in other environmental sample types should be examined in future studies.

## Figures and Tables

**Table 1:** Saccharibacteria-specific primers targeting the 16S rRNA gene evaluated in this study.

Primer	Sequence (5′ to 3′)	Reference
TM7314F	GAGAGGATGATCAGCCAG	[10]
TM7580F	AYTGGGCGTAAAGAGTTGC	[10]
Sac1031F	AAGAGAACTGTGCCTTCGG	[17]
400F	TATGAGTGAAGAATATGAC	[13]
TM7-910R	GTCCCCGTCAATTCCTTTATG	[16]
TM7-1177R	GACCTGACATCATCCCCTCCTTCC	[16]
Sac1218R	GCGTAAGGGAAATACTGACC	[17]
1110R	CAGTCCAAGTAGAAAAATAC	[13]

**Table 2:** Annealing temperature and specificity of Saccharibacteria-specific primer sets tested in this study.

Primer Set	Expected Fragment Length (bp)	DNA Band	Annealing Temperature (°C)	Number of Saccharibacteria Sequences ^a^/Number of Total Clones Analyzed (Specificity)
TM7314F and TM7-910R	596	yes	64	95/95 (100%)
TM7314F and TM7-1177R	863	yes	64	96/96 (100%)
TM7580F and TM7-910R	330	yes	66	94/94 (100%)
TM7580F and TM7-1177R	597	yes	63	94/94 (100%)
Sac1031F and Sac1218R	187	no	Not determined	Not determined
400F and 1100R	700	no	Not determined	Not determined

^a^ Saccharibacteria sequences were confirmed with a basic local alignment search tool (BLAST) search [19] and the ARB software [20].

**Table 3:** Copy number of Saccharibacteria 16S rRNA gene as determined by qPCR.

Primer Set	Copy Number of Plasmid (Copies/ng-DNA) ^a^	Measured Copy Number of Plasmid (Copies/ng-DNA) ^b^	Copy Number of Saccharibacteria in Activated Sludge (Copies/g-MLSS) ^b^
TM7314F and TM7-910R	1.0 × 10^3^	1.4 ± 0.1 × 10^3^	6.8 ± 0.3 × 10^6^
TM7314F and TM7-1177R	9.6 × 10^3^	1.4 ± 0.1 × 10^4^	5.6 ± 1.1 × 10^6^
TM7580F and TM7-910R	8.9 × 10^3^	1.8 ± 0.2 × 10^4^	6.7 ± 1.1 × 10^7^
TM7580F and TM7-1177R	9.9 × 10^4^	2.1 ± 0.3 × 10^5^	3.5 ± 1.0 × 10^7^

^a^ Values are calculated from the known copy number of plasmid DNA carrying Saccharibacteria 16S rRNA genes extracted from the four clone libraries; ^b^ Values are means ± standard deviations (*n* = 3); MLSS, mixed liquor suspended solids.

**Table 4:** Sequence mismatches of the primers and the targeted 16S rRNA gene region of *Hydrogenophaga* sp.

Primer	Sequence of *Hydrogenophaga* sp. (5′ to 3′) ^a^
TM7314F	GAGAGGACGACCAGCCAC
TM7580F	ACTGGGCGTAAAGCGTGCG
TM7-910R	CTCAAAGGAATTGACGGGGAC
TM7-1177R	GGAAGGTGGGGATGACGTCAAGTC

^a^ Red represents mismatch in *Hydrogenophaga* sp. sequence targeted by the Saccharibacteria primers.

## Supplementary Materials

The following are available online at http://www.mdpi.com/1996-1944/11/7/1129/s1, Figure S1: Primer-target sequence mismatches of Saccharibacteria-specific primers evaluated in this study, Figure S2: Concentration of PCR product using the primer set with TM7314F and TM7-910R (**A**), TM7314F and TM7-1177R (**B**), TM7580F and TM7-910R (**C**), TM7580F and TM7-1177R (**D**), Sac1031F and Sac1218R (**E**), and 400F and 1110R (**F**), Figure S3: Phylogenetic tree of 1303 Saccharibacteria sequences and related operational taxonomic units (OTUs) obtained from activated sludge, Figure S4: Standard curves of Saccharibacteria qPCR for the measurement of copy number of plasmids using 10-fold serial dilutions of plasmid DNA carrying Saccharibacteria 16S rRNA genes and the four primer sets, Figure S5: Melting curves of the four standard curves using 10-fold serial dilutions of plasmid DNA carrying Saccharibacteria 16S rRNA genes and the four primer sets, Table S1: Phylogenetic relatives of the OTUs analyzed in this study. Please add the information.
