# Supplementary material for: Specificities and Efficiencies of Primers Targeting Candidatus Phylum Saccharibacteria in Activated Sludge
Source: Materials (Basel). 2018 Jul 3;11(7):1129. doi: 10.3390/ma11071129 (PMC6073563; doi:10.3390/ma11071129)
Supplement: Supplementary file 1 [file materials-11-01129-s001.pdf]

# Specificities and Efficiencies of Primers Targeting *Candidatus* Phylum Saccharibacteria in Activated Sludge

**Ryota Takenaka, Yoshiteru Aoi, Noriatsu Ozaki, Akiyoshi Ohashi, and Tomonori Kindaichi**

Supplementary Materials:

(A) Primer TM7314F

| Target (5'-3') | G | A | G | A | G | G | A | T | G | A | T | C | A | G | C | C | A | G | |
|---|---|---|---|---|---|---|---|---|---|---|---|---|---|---|---|---|---|---|---|
| A | 0 | 1302 | 3 | 1298 | 26 | 4 | 1263 | 3 | 1 | 1294 | 5 | 0 | 1284 | 3 | 1 | 0 | 1251 | 1 | |
| T | 1 | 0 | 0 | 0 | 1 | 0 | 30 | 1294 | 0 | 0 | 1050 | 0 | 1 | 98 | 191 | 0 | 49 | 1 | |
| G | 1300 | 0 | 1296 | 4 | 1274 | 1296 | 9 | 0 | 1301 | 7 | 0 | 0 | 6 | 1198 | 1 | 1 | 2 | 1143 | |
| C | 1 | 0 | 3 | 1 | 2 | 1 | 1 | 4 | 0 | 1 | 246 | 1303 | 12 | 3 | 1110 | 1302 | 1 | 158 | |
| Other* | 1 | 1 | 1 | 0 | 0 | 2 | 0 | 2 | 1 | 1 | 2 | 0 | 0 | 1 | 0 | 0 | 0 | 0 | Average |
| Coverage (%) | 99 | 99 | 99 | 99 | 97 | 99 | 96 | 99 | 99 | 99 | 80 | 100 | 98 | 91 | 85 | 99 | 96 | 87 | 95.6% |

(B) Primer TM7580F

| Target (5'-3') | A | Y | T | G | G | G | C | G | T | A | A | A | G | A | G | T | T | G | C | |
|---|---|---|---|---|---|---|---|---|---|---|---|---|---|---|---|---|---|---|---|---|
| A | 1301 | 1 | 1 | 0 | 2 | 1 | 0 | 1 | 0 | 1301 | 1302 | 1298 | 3 | 1237 | 0 | 10 | 1 | 5 | 1 | |
| T | 0 | 16 | 1300 | 0 | 0 | 1 | 96 | 0 | 1300 | 1 | 0 | 0 | 0 | 1 | 5 | 1292 | 1240 | 7 | 28 | |
| G | 2 | 0 | 1 | 1303 | 1301 | 1298 | 1 | 1300 | 1 | 0 | 1 | 1 | 1300 | 3 | 1298 | 0 | 1 | 1285 | 1 | |
| C | 0 | 1286 | 1 | 0 | 0 | 2 | 1206 | 2 | 1 | 0 | 0 | 0 | 0 | 62 | 0 | 1 | 3 | 6 | 1273 | |
| Other* | 0 | 0 | 0 | 0 | 0 | 1 | 0 | 0 | 1 | 3 | 0 | 4 | 0 | 0 | 0 | 0 | 58 | 0 | 0 | Average |
| Coverage (%) | 99 | 98 | 99 | 100 | 99 | 99 | 92 | 99 | 99 | 99 | 99 | 99 | 99 | 94 | 99 | 99 | 95 | 98 | 97 | 98.0% |

(C) Primer Sac1031F

| Target (5'-3') | A | A | G | A | G | A | A | C | T | G | T | G | C | C | T | T | C | G | G | |
|---|---|---|---|---|---|---|---|---|---|---|---|---|---|---|---|---|---|---|---|---|
| A | 1298 | 1302 | 2 | 194 | 944 | 312 | 993 | 135 | 669 | 0 | 1 | 18 | 48 | 12 | 9 | 1 | 491 | 57 | 101 | |
| T | 0 | 0 | 65 | 238 | 22 | 62 | 214 | 142 | 568 | 30 | 1292 | 0 | 33 | 522 | 1195 | 1267 | 409 | 791 | 472 | |
| G | 5 | 0 | 1145 | 643 | 136 | 926 | 82 | 674 | 31 | 1263 | 5 | 1229 | 1 | 14 | 53 | 3 | 18 | 409 | 726 | |
| C | 0 | 0 | 91 | 228 | 201 | 2 | 12 | 352 | 35 | 3 | 3 | 47 | 1211 | 749 | 45 | 31 | 330 | 5 | 2 | |
| Other* | 0 | 1 | 0 | 0 | 0 | 1 | 2 | 0 | 0 | 7 | 2 | 9 | 10 | 6 | 1 | 1 | 55 | 41 | 2 | Average |
| Coverage (%) | 99 | 99 | 87 | 14 | 10 | 23 | 76 | 27 | 43 | 96 | 99 | 94 | 92 | 57 | 91 | 97 | 25 | 31 | 55 | 63.9% |

(D) Primer 400F

| Target (5'-3') | T | A | T | G | A | G | T | G | A | A | G | A | A | T | A | T | G | A | C | |
|---|---|---|---|---|---|---|---|---|---|---|---|---|---|---|---|---|---|---|---|---|
| A | 0 | 1148 | 29 | 631 | 739 | 34 | 45 | 0 | 1300 | 796 | 93 | 1300 | 865 | 35 | 1180 | 11 | 1 | 1299 | 1 | |
| T | 1301 | 15 | 1264 | 169 | 504 | 21 | 1005 | 0 | 1 | 51 | 3 | 2 | 407 | 1266 | 113 | 1276 | 0 | 0 | 91 | |
| G | 0 | 74 | 3 | 324 | 47 | 1217 | 70 | 1301 | 2 | 227 | 1206 | 0 | 5 | 1 | 4 | 2 | 1301 | 3 | 0 | |
| C | 2 | 66 | 7 | 179 | 12 | 31 | 182 | 1 | 0 | 228 | 0 | 0 | 24 | 1 | 5 | 14 | 0 | 0 | 1211 | |
| Other* | 0 | 0 | 0 | 0 | 1 | 0 | 1 | 1 | 0 | 1 | 1 | 1 | 2 | 0 | 1 | 0 | 1 | 1 | 0 | Average |
| Coverage (%) | 99 | 88 | 97 | 24 | 56 | 93 | 77 | 99 | 99 | 61 | 92 | 99 | 66 | 97 | 90 | 97 | 99 | 99 | 92 | 85.5% |

(E) Primer TM7-910R

| Target (5'-3') | C | A | T | A | A | A | G | G | A | A | T | T | G | A | C | G | G | G | G | A | C | |
|---|---|---|---|---|---|---|---|---|---|---|---|---|---|---|---|---|---|---|---|---|---|---|
| A | 0 | 1156 | 0 | 1302 | 1302 | 1299 | 0 | 0 | 1301 | 1300 | 0 | 6 | 0 | 1302 | 0 | 3 | 2 | 1 | 1 | 1286 | 2 | |
| T | 0 | 143 | 1164 | 0 | 0 | 0 | 0 | 0 | 0 | 0 | 1300 | 1293 | 0 | 1 | 0 | 1 | 1 | 1 | 0 | 3 | 24 | |
| G | 0 | 1 | 0 | 1 | 1 | 3 | 1303 | 1303 | 1 | 3 | 0 | 1 | 1303 | 0 | 0 | 1299 | 1299 | 1301 | 1302 | 4 | 26 | |
| C | 1303 | 2 | 139 | 0 | 0 | 0 | 0 | 0 | 0 | 0 | 1 | 3 | 0 | 0 | 1303 | 0 | 1 | 0 | 0 | 9 | 1250 | |
| Other* | 0 | 1 | 0 | 0 | 0 | 1 | 0 | 0 | 1 | 0 | 2 | 0 | 0 | 0 | 0 | 0 | 0 | 0 | 0 | 1 | 1 | Average |
| Coverage (%) | 100 | 88 | 89 | 99 | 99 | 99 | 100 | 100 | 99 | 99 | 99 | 99 | 100 | 99 | 100 | 99 | 99 | 99 | 99 | 98 | 95 | 98.0% |

(F) Primer TM7-1177R

| Target (5'-3') | G | G | A | A | G | G | A | G | G | G | G | A | T | G | A | T | G | T | C | A | G | G | T | C | |
|---|---|---|---|---|---|---|---|---|---|---|---|---|---|---|---|---|---|---|---|---|---|---|---|---|---|
| A | 2 | 2 | 1303 | 1290 | 1 | 2 | 1101 | 4 | 5 | 0 | 0 | 1294 | 5 | 1 | 1303 | 16 | 2 | 2 | 0 | 1274 | 6 | 1 | 0 | 0 | |
| T | 1 | 0 | 0 | 2 | 0 | 1 | 49 | 0 | 1 | 0 | 0 | 5 | 1256 | 1 | 0 | 1106 | 4 | 1283 | 5 | 24 | 1 | 0 | 1299 | 0 | |
| G | 1299 | 1301 | 0 | 6 | 1301 | 1295 | 146 | 1298 | 1297 | 1302 | 1302 | 3 | 3 | 1301 | 0 | 30 | 1297 | 0 | 1 | 1 | 1294 | 1301 | 0 | 0 | |
| C | 0 | 0 | 0 | 2 | 1 | 2 | 3 | 0 | 0 | 0 | 0 | 1 | 39 | 0 | 0 | 150 | 0 | 14 | 1297 | 4 | 2 | 0 | 4 | 1303 | |
| Other* | 1 | 0 | 0 | 3 | 0 | 3 | 4 | 1 | 0 | 1 | 1 | 0 | 0 | 0 | 0 | 1 | 0 | 4 | 0 | 0 | 0 | 1 | 0 | 0 | Average |
| Coverage (%) | 99 | 99 | 100 | 99 | 99 | 99 | 84 | 99 | 99 | 99 | 99 | 99 | 96 | 99 | 100 | 84 | 99 | 98 | 99 | 97 | 99 | 99 | 99 | 100 | 97.6% |

(G) Primer Sac1218R

| Target (5'-3') | G | G | T | C | A | G | T | A | T | T | T | C | C | C | T | T | A | C | G | C | |
|---|---|---|---|---|---|---|---|---|---|---|---|---|---|---|---|---|---|---|---|---|---|
| A | 6 | 1 | 0 | 0 | 1201 | 2 | 0 | 1300 | 0 | 0 | 394 | 6 | 0 | 1 | 1 | 0 | 1298 | 1 | 461 | 3 | |
| T | 1 | 0 | 1299 | 0 | 32 | 53 | 1235 | 1 | 1302 | 1214 | 714 | 4 | 176 | 4 | 1297 | 1299 | 1 | 60 | 0 | 849 | |
| G | 1294 | 1301 | 2 | 0 | 29 | 1248 | 0 | 2 | 0 | 68 | 194 | 46 | 11 | 1 | 1 | 0 | 2 | 0 | 839 | 1 | |
| C | 2 | 0 | 2 | 1303 | 41 | 0 | 67 | 0 | 1 | 20 | 1 | 1247 | 1115 | 1296 | 3 | 4 | 2 | 1242 | 3 | 449 | |
| Other* | 0 | 1 | 0 | 0 | 0 | 0 | 1 | 0 | 0 | 1 | 0 | 0 | 1 | 1 | 1 | 0 | 0 | 0 | 0 | 1 | Average |
| Coverage (%) | 99 | 99 | 99 | 100 | 92 | 95 | 94 | 99 | 99 | 93 | 54 | 95 | 85 | 99 | 99 | 99 | 99 | 95 | 64 | 34 | 89.6% |

(H) Primer 1110R

| Target (5'-3') | G | T | A | T | T | T | T | T | C | T | A | C | T | T | G | G | A | C | T | G | |
|---|---|---|---|---|---|---|---|---|---|---|---|---|---|---|---|---|---|---|---|---|---|
| A | 21 | 306 | 1296 | 6 | 24 | 2 | 2 | 1 | 0 | 109 | 1035 | 279 | 116 | 109 | 1111 | 71 | 1301 | 0 | 2 | 0 | |
| T | 2 | 702 | 4 | 1291 | 1269 | 1291 | 1142 | 1300 | 0 | 1191 | 30 | 327 | 756 | 426 | 0 | 18 | 0 | 1 | 1260 | 1 | |
| G | 1273 | 289 | 0 | 1 | 2 | 1 | 28 | 0 | 1 | 0 | 227 | 563 | 29 | 147 | 188 | 1213 | 2 | 0 | 1 | 1302 | |
| C | 0 | 4 | 0 | 5 | 3 | 1 | 127 | 2 | 1301 | 3 | 11 | 133 | 402 | 620 | 4 | 0 | 0 | 1301 | 40 | 0 | |
| Other* | 7 | 2 | 3 | 0 | 5 | 8 | 4 | 0 | 1 | 0 | 0 | 1 | 0 | 1 | 0 | 1 | 0 | 1 | 0 | 0 | Average |
| Coverage (%) | 97 | 53 | 99 | 99 | 97 | 99 | 87 | 99 | 99 | 91 | 79 | 10 | 58 | 32 | 14 | 93 | 99 | 99 | 96 | 99 | 80.0% |

**Figure S1.** Primer-target sequence mismatches of Saccharibacteria-specific primers evaluated in this study. In total, 1303 Saccharibacteria sequences were retrieved from the SILVA SSU 132 database. Different bases in the position were counted, and coverages at the position are indicated with red for >90% coverage, orange for >75% coverage, and yellow for >50% coverage. *, mixed-base or gap

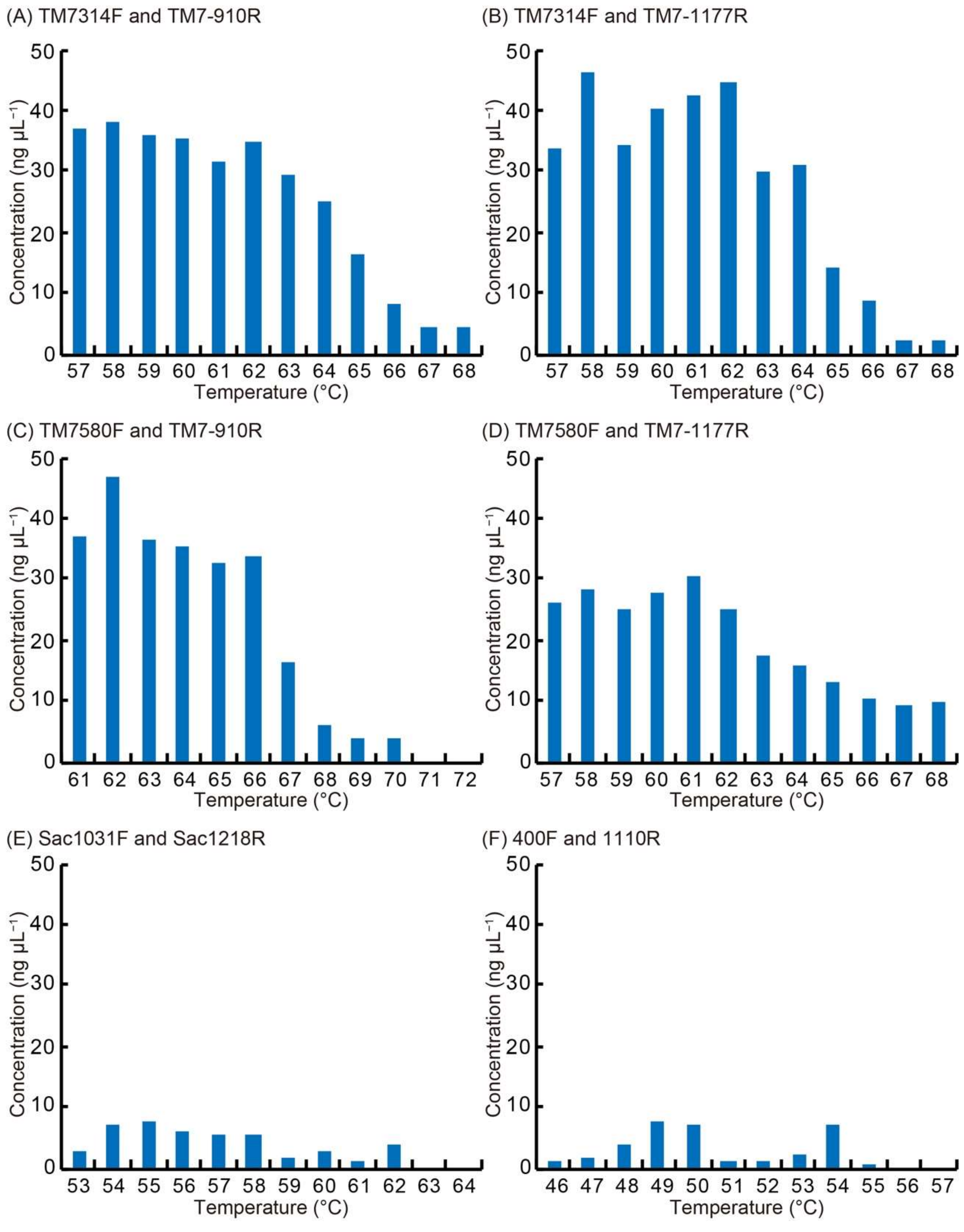


**Figure S2.** Concentration of PCR product using the primer set with TM7314F and TM7-910R (**A**), TM7314F and TM7-1177R (**B**), TM7580F and TM7-910R (**C**), TM7580F and TM7-1177R (**D**), Sac1031F and Sac1218R (**E**), and 400F and 1110R (**F**).

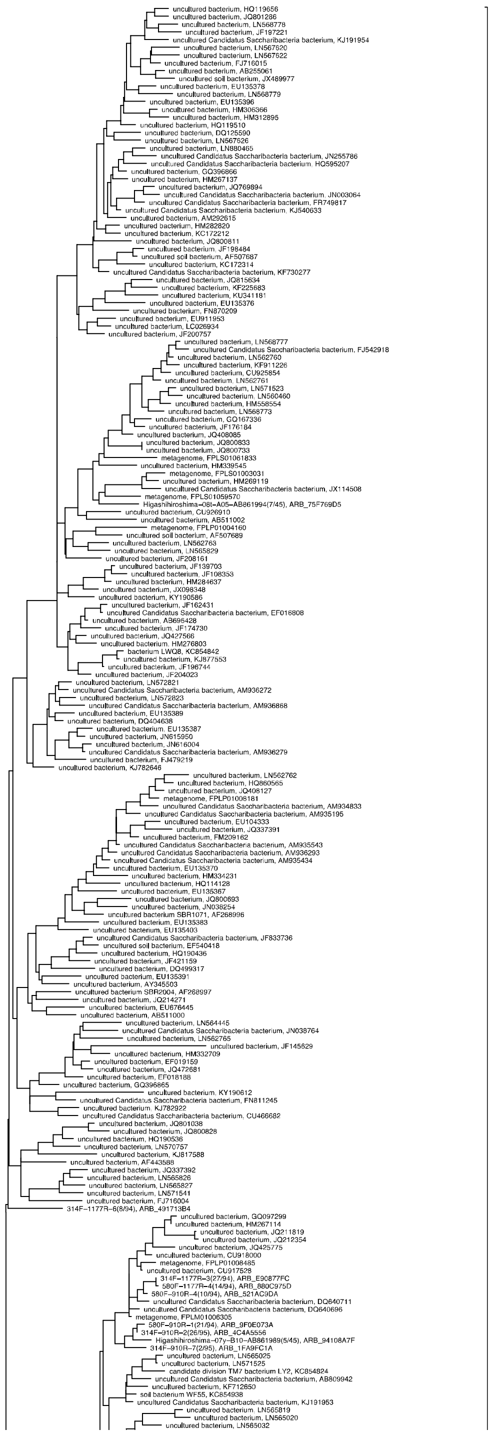

**Figure S3.** Phylogenetic tree of 1,303 Saccharibacteria sequences and related operational taxonomic units (OTUs) obtained from activated sludge. *Thermotoga* sequences were used as an outgroup.

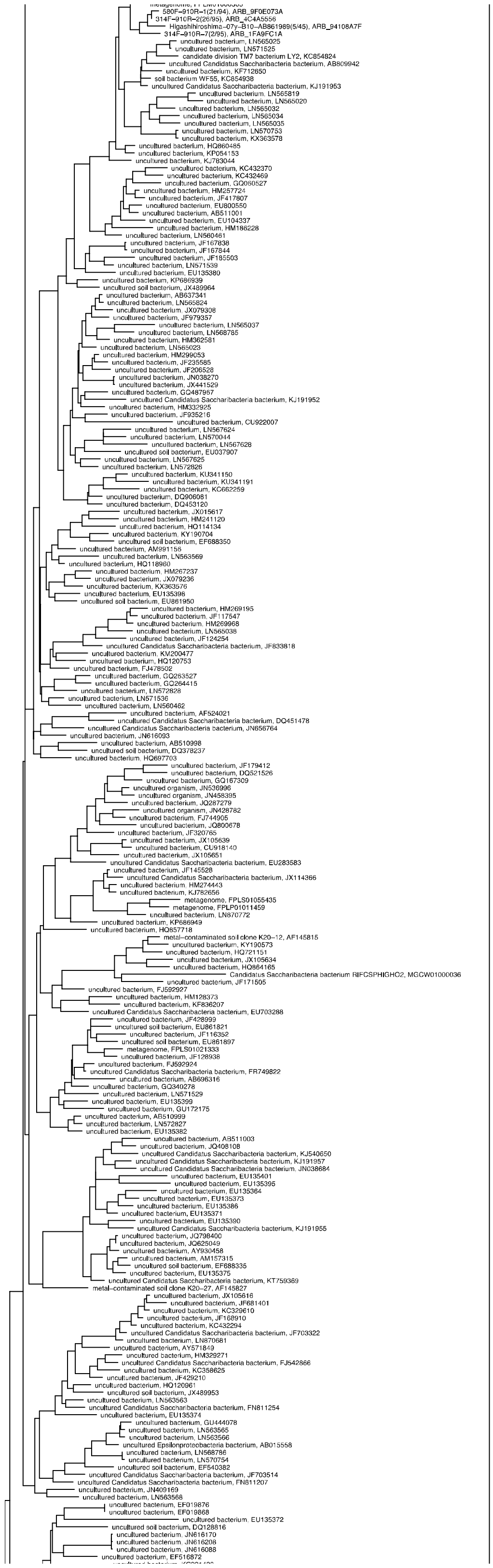

**Figure S3.** Continued.

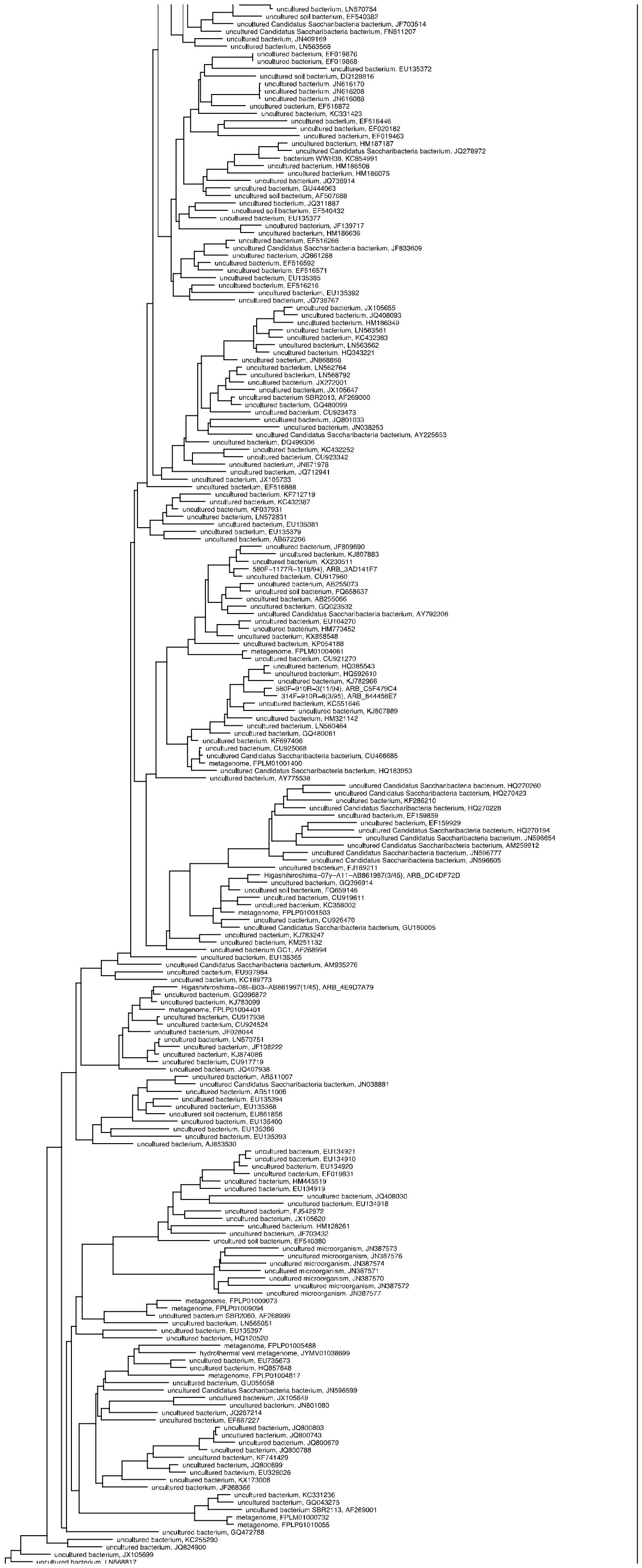

**Figure S3.** Continued.

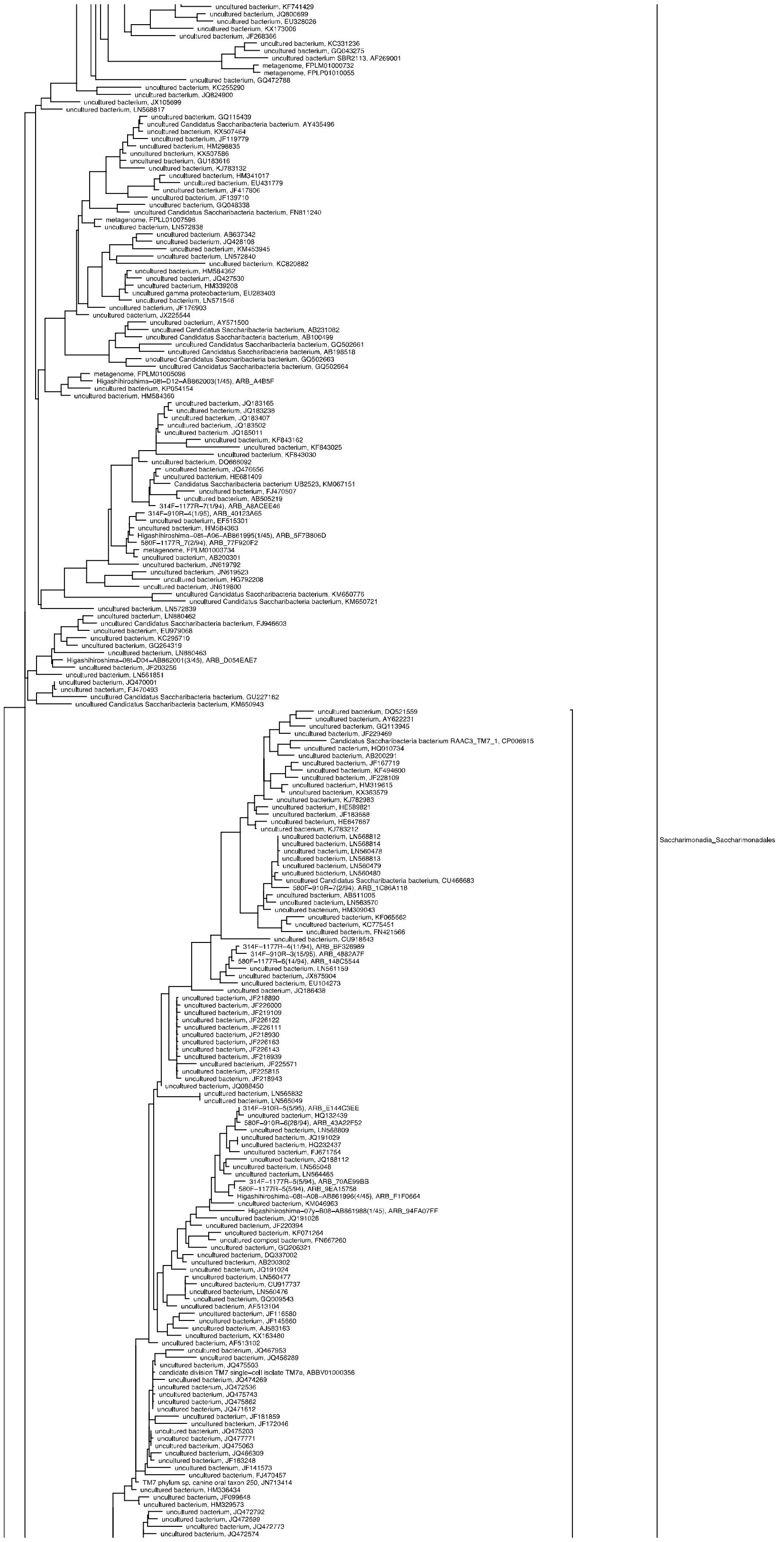


**Figure S3.** Continued.

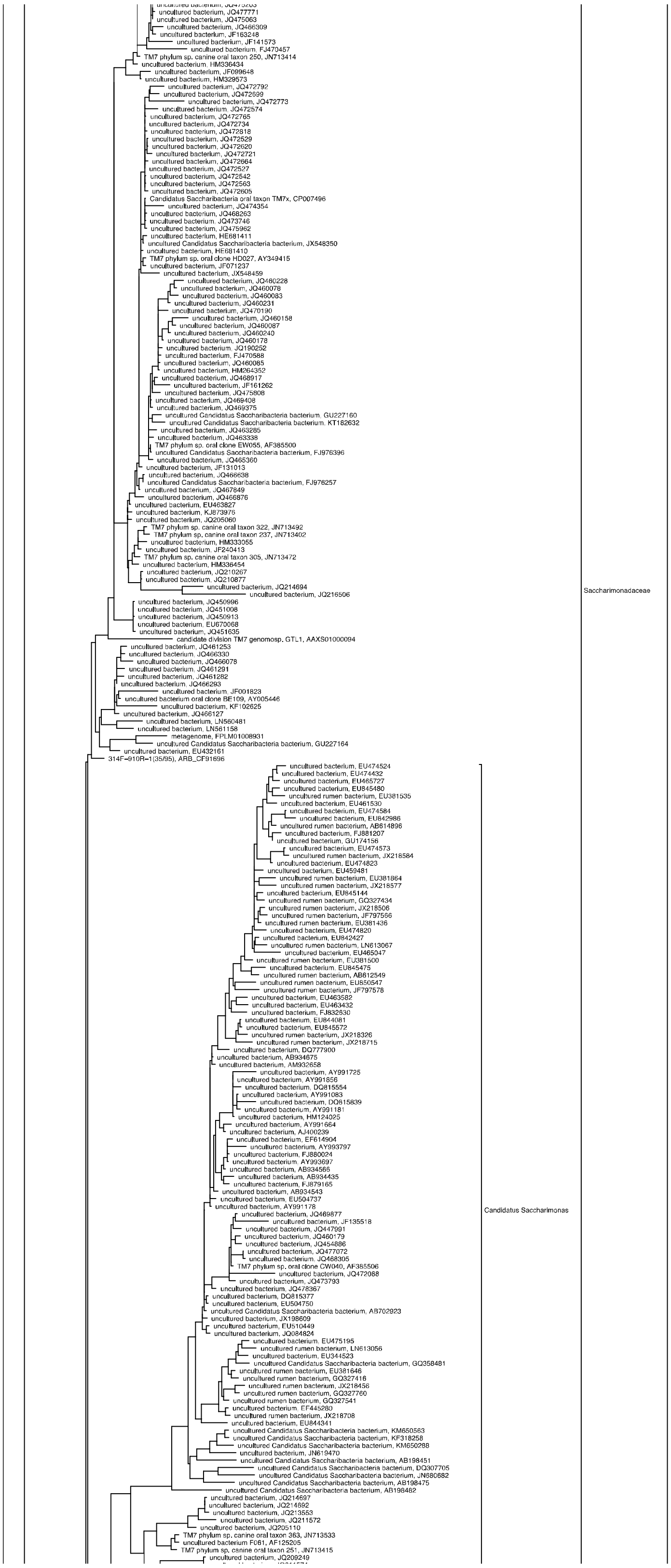


**Figure S3.** Continued.

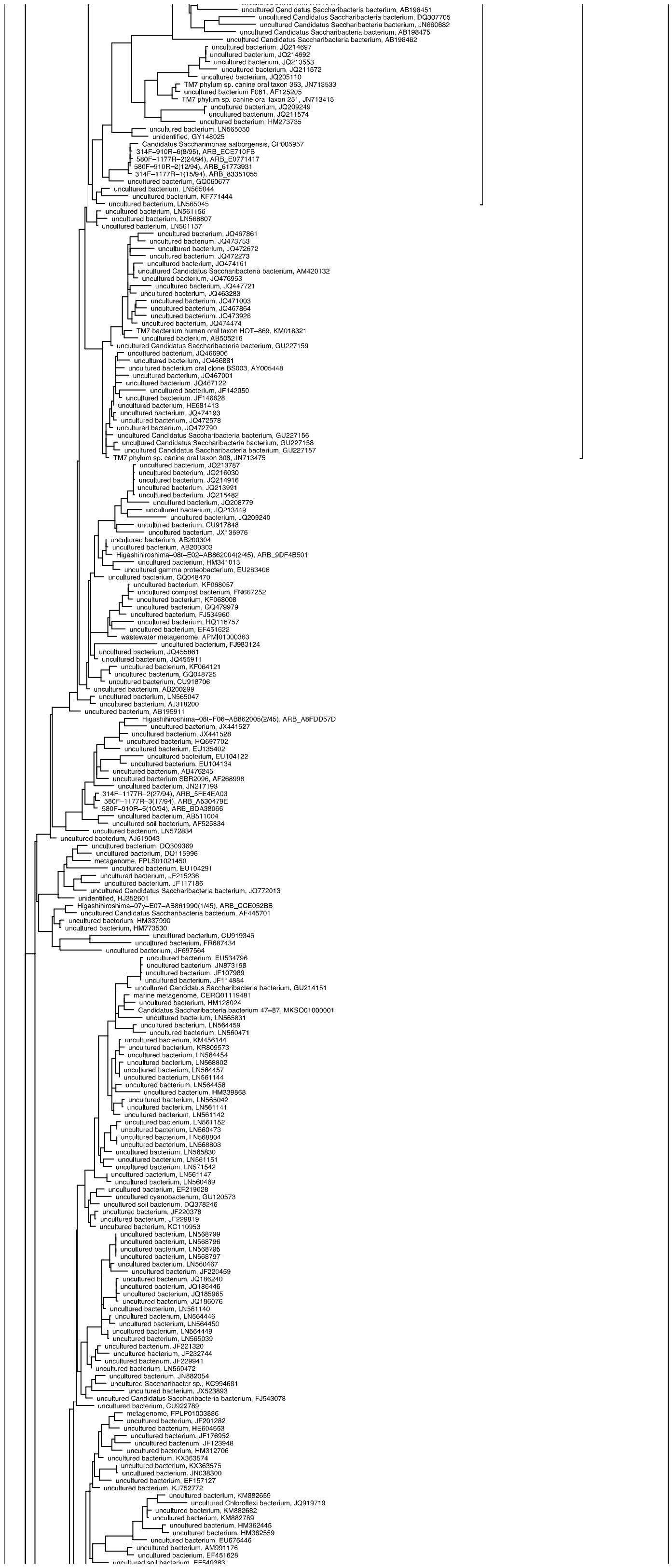

**Figure S3.** Continued.

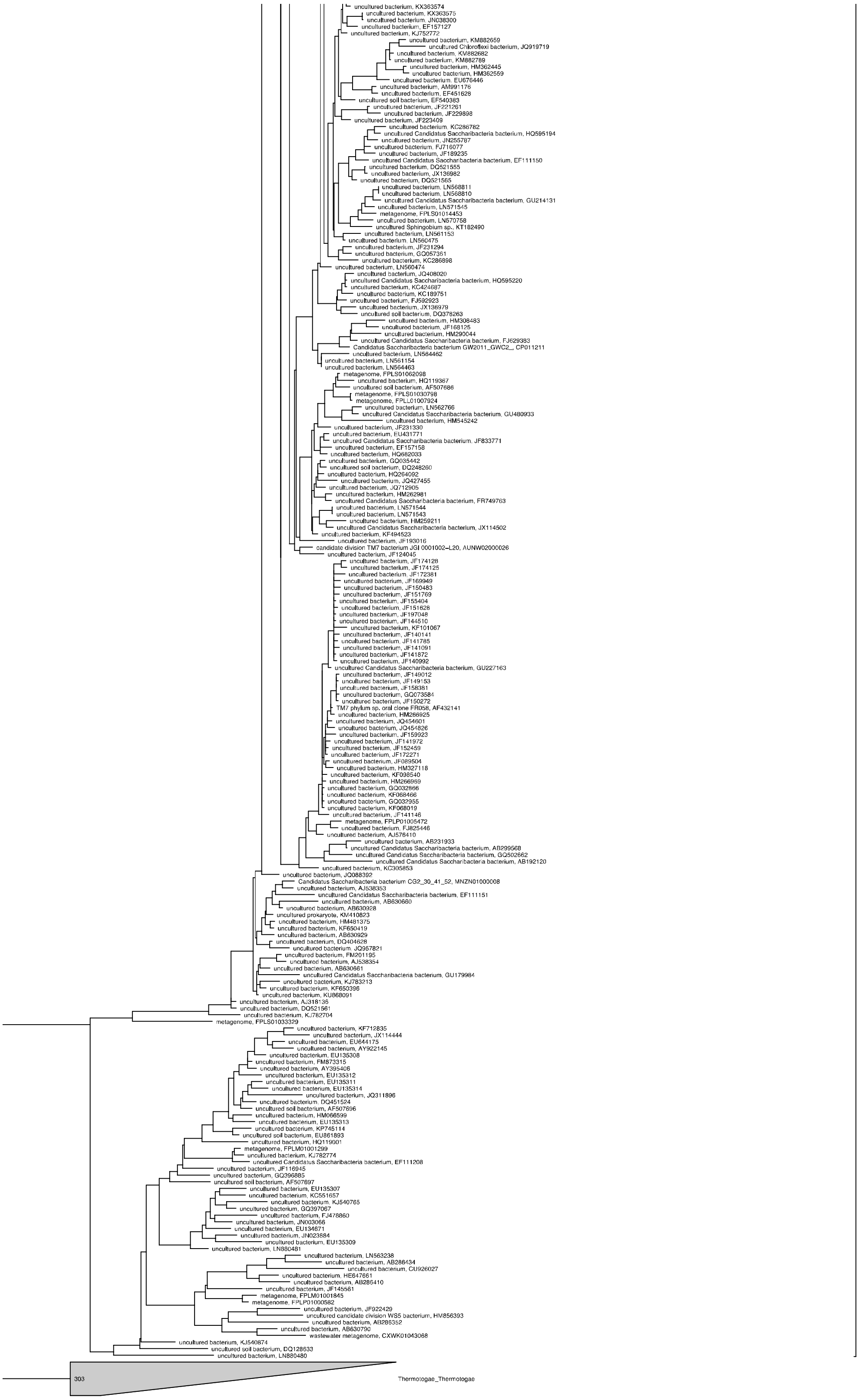


**Figure S3.** Continued.

**Table S1.** Phylogenetic relatives of the OTUs analyzed in this study.

| OTU | Closest relatives | Accession No. | Identity (%) |
|---|---|---|---|
| TM7314F–TM7-910R_1 (35/95) | Uncultured bacterium | EU432161 | 96.7 |
| TM7314F–TM7-910R_ 2 (26/95) | Metagenome | FPLM01006305 | 96.6 |
| TM7314F–TM7-910R_3 (15/95) | Uncultured bacterium | JX875904 | 97.4 |
| TM7314F–TM7-910R_4 (1/95) | Uncultured bacterium | EF515301 | 97.7 |
| TM7314F–TM7-910R_5 (5/95) | Uncultured bacterium | HQ132439 | 99.8 |
| TM7314F–TM7-910R_6 (8/95) | *Candidatus* Saccharimonas aalborgensis | CP005957 | 99.8 |
| TM7314F–TM7-910R_7 (2/95) | metagenome | FPLM01006305 | 96.6 |
| TM7314F–TM7-910R_8 (3/95) | Uncultured bacterium | HQ385543 | 95.7 |
| TM7314F–TM7-1177R_1 (15/94) | *Candidatus* Saccharimonas aalborgensis | CP005957 | 99.7 |
| TM7314F–TM7-1177R_2 (27/94) | uncultured soil bacterium | AF525834 | 95.0 |
| TM7314F–TM7-1177R_3 (27/94) | Uncultured *Candidatus* Saccharibacteria bacterium | DQ640711 | 98.6 |
| TM7314F–TM7-1177R_4 (11/94) | Uncultured bacterium | JX875904 | 98.5 |
| TM7314F–TM7-1177R_5 (5/94) | Uncultured bacterium | KM046963 | 94.3 |
| TM7314F–TM7-1177R_6 (8/94) | Uncultured bacterium | LN571536 | 92.6 |
| TM7314F–TM7-1177R_7 (1/94) | Uncultured bacterium | JQ476656 | 99.3 |
| TM7580F–TM7-910R_1 (21/94) | Metagenome | FPLM01006305 | 94.3 |
| TM7580F–TM7-910R_2 (12/94) | *Candidatus* Saccharimonas aalborgensis | CP005957 | 100.0 |
| TM7580F–TM7-910R_3 (11/94) | Uncultured bacterium | HQ385543 | 95.4 |
| TM7580F–TM7-910R_4 (10/94) | Uncultured Candidatus Saccharibacteria bacterium | DQ640711 | 98.9 |
| TM7580F–TM7-910R_5 (10/94) | Uncultured soil bacterium | AF525834 | 96.5 |
| TM7580F–TM7-910R_6 (28/94) | Uncultured bacterium | HQ132439 | 99.5 |
| TM7580F–TM7-910R_7 (2/94) | Uncultured bacterium | LN568812 | 97.0 |
| TM7580F–TM7-1177R_1 (18/94) | Uncultured bacterium | CU917960 | 97.4 |
| TM7580F–TM7-1177R_2 (24/94) | *Candidatus* Saccharimonas aalborgensis | CP005957 | 99.8 |
| TM7580F–TM7-1177R_3 (17/94) | Uncultured soil bacterium | AF525834 | 93.6 |
| TM7580F–TM7-1177R_4 (14/94) | Uncultured *Candidatus* Saccharibacteria bacterium | DQ640711 | 98.6 |
| TM7580F–TM7-1177R_5 (5/94) | Uncultured bacterium | KM046963 | 93.9 |
| TM7580F–TM7-1177R_6 (14/94) | Uncultured bacterium | JX875904 | 98.4 |
| TM7580F–TM7-1177R_7 (2/94) | Uncultured bacterium | HM584363 | 98.7 |

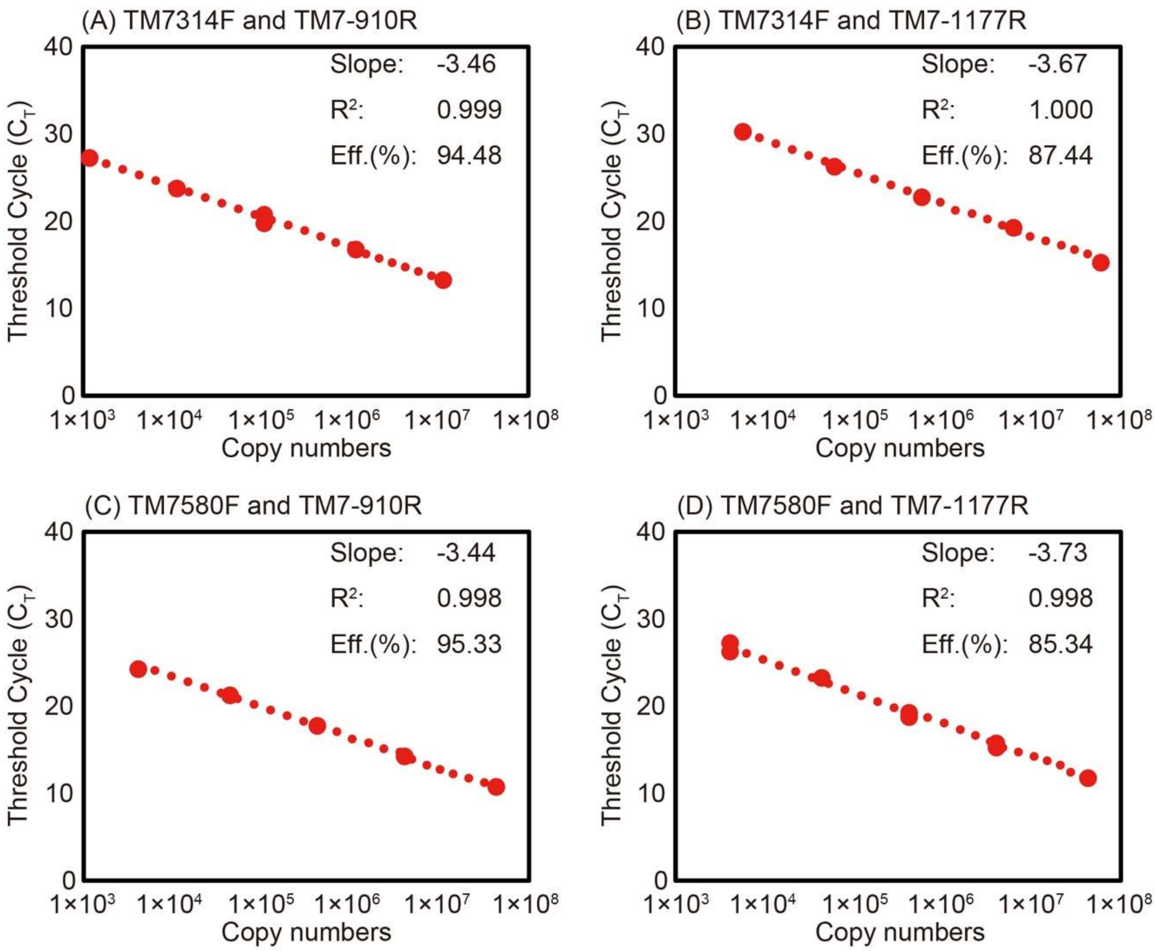


**Figure S4.** Standard curves of Saccharibacteria qPCR for the measurement of copy number of plasmids using 10-fold serial dilutions of plasmid DNA carrying Saccharibacteria 16S rRNA genes and the four primer sets: TM7314F and TM7-910R (**A**); TM7314F and TM7-1177R (**B**); TM7580F and TM7-910R (**C**); and TM7580F and TM7-1177R (**D**). The slope, coefficient of determination ($R^2$), and amplification efficiency were also shown in the figures.

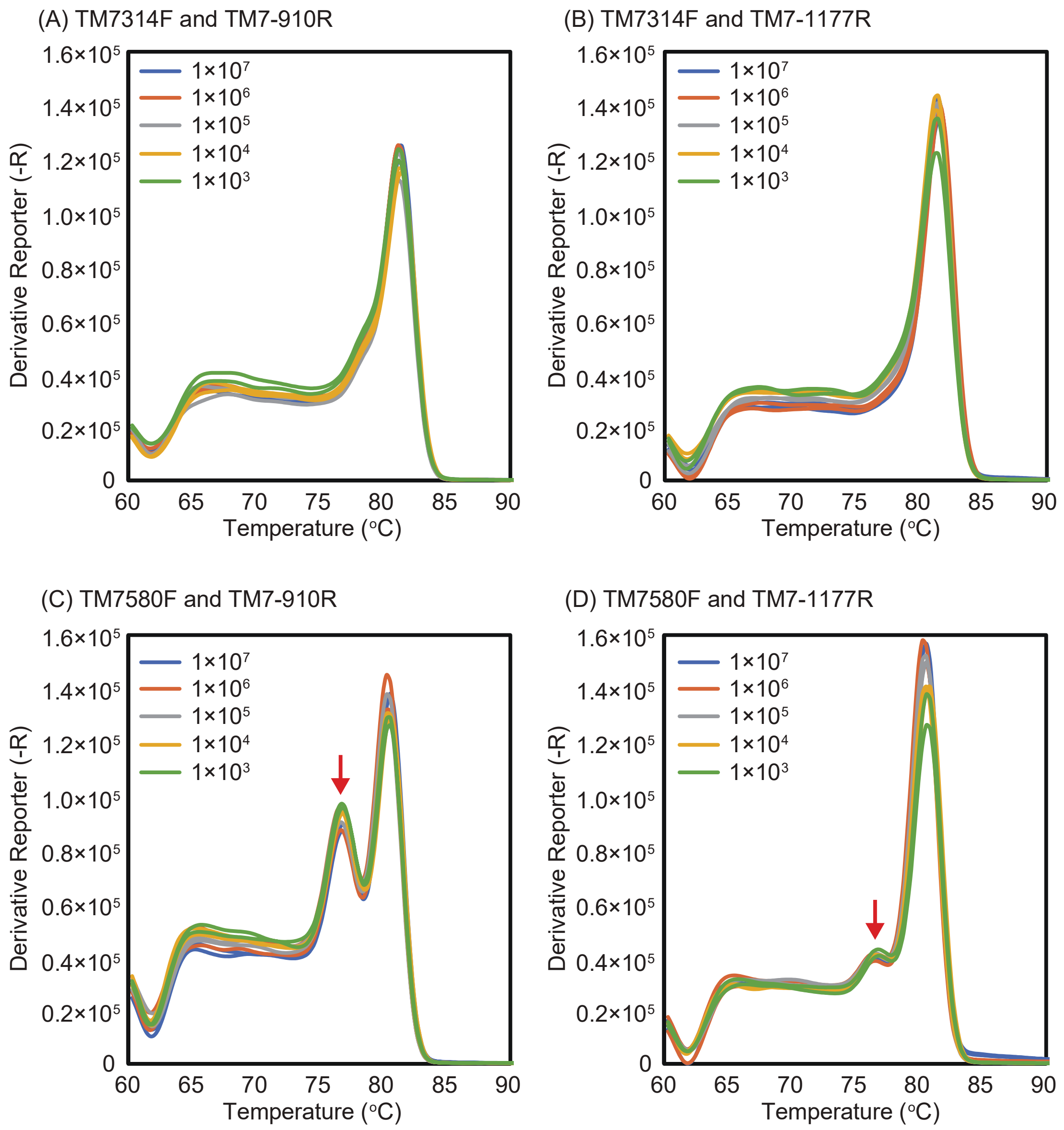


**Figure S5.** Melting curves of the four standard curves using 10-fold serial dilutions of plasmid DNA carrying Saccharibacteria 16S rRNA genes and the four primer sets: TM7314F and TM7-910R (**A**); TM7314F and TM7-1177R (**B**); TM7580F and TM7-910R (**C**); and TM7580F and TM7-1177R (**D**). Red arrows indicate a minor peak, suggesting the presence of non-specific amplification products. In addition to the main peak, another peak at around 77 °C was observed when TM7580F was used (red arrows in (C) and (D)), suggesting the presence of non-specific amplification products, specifically short products, which were not expected from these primer sets.
